# Period of hospitalization and mortality in transferred versus non-transferred COVID-19 patients: results from Germany

**DOI:** 10.1038/s41598-024-57272-y

**Published:** 2024-03-28

**Authors:** Pascal Suski, Rudolf A. Jörres, Sebastian Engelhardt, Kathrin Kahnert, Katharina Lenherr, Andreas Bauer, Stephan Budweiser

**Affiliations:** 1 Department of Internal Medicine III, Division of Pneumology and Respiratory Medicine, RoMed Hospital Rosenheim, Ellmaierstraße 23, 83022 Rosenheim, Germany; 2 Institute and Outpatient Clinic for Occupational, Social and Environmental Medicine, LMU University Hospital, LMU Munich, Comprehensive Pneumology Center Munich (CPC-M), Member of the German Center for Lung Research (DZL), Ziemssenstraße 1, 80336 Munich, Germany; 3 Department of Emergency, RoMed Hospital Rosenheim, Ellmaierstraße 23, 83022 Rosenheim, Germany; 4 Department of Medicine V, LMU University Hospital, LMU Munich, Comprehensive Pneumology Center, Member of the German Center for Lung Research (DZL), Marchioninistraße 15, 80336 Munich, Germany; 5 Pneumological Center, MediCenter Germering, Hartstraße 52, 82110 Munich, Germany; 6 Institute for Anesthesiology and Surgical Intensive Care Medicine, RoMed Hospital Rosenheim, Ellmaierstraße 23, 83022 Rosenheim, Germany; 7 University Hospital Regensburg, Franz-Josef-Strauß-Allee 11, 93053 Regensburg, Germany

**Keywords:** Viral infection, Health services

## Abstract

COVID-19 was a challenge for health-care systems worldwide, causing large numbers of hospitalizations and inter-hospital transfers. We studied whether transfer, as well as its reason, was associated with the duration of hospitalization in non-ICU and ICU patients. For this purpose, all patients hospitalized due to COVID-19 between August 1st and December 31st, 2021, in a network of hospitals in Southern Germany were comprehensively characterized regarding their clinical course, therapy, complications, transfers, reasons for transfer, involved levels of care, total period of hospitalization and in-hospital mortality, using univariate and multiple regression analyses. While mortality was not significantly associated with transfer, the period of hospitalization was. In non-ICU patients (*n* = 545), median (quartiles) time was 7.0 (4.0–11.0) in non-transferred (*n* = 458) and 18.0 (11.0–29.0) days in transferred (*n* = 87) patients (*p* < 0.001). In ICU patients (*n* = 100 transferred, *n* = 115 non-transferred) it was 12.0 (8.3–18.0) and 22.0 (15.0–34.0) days (*p* < 0.001). Beyond ECMO therapy (4.5%), reasons for transfer were medical (33.2%) or capacity (61.9%) reasons, with medical/capacity reasons in 32/49 of non-ICU and 21/74 of ICU patients. Thus, the transfer of COVID-19 patients between hospitals was associated with longer periods of hospitalization, corresponding to greater health care utilization, for which specific patient characteristics and clinical decisions played a role.

## Introduction

With the widespread SARS-Cov-2 infections in the beginning of 2020, COVID-19 appeared as an outstanding challenge for health-care worldwide, which was particularly reflected in the numbers of hospitalized patients and mortality attributed to the SARS-CoV-2 infection^1,2^. COVID-19 exerted considerable stress on medical capacity (e.g., hospital beds and medical staff^3,4^, requiring a sophisticated patient management that included optimal allocation to the available hospital capacity and capability^5^. In Germany, as in other countries, this led to a high number of inter-hospital transfers^5–9^, particularly regarding intensive care treatment, raising the question whether the transfers had an impact on patient outcomes.

Although mortality from COVID-19 decreased over time^10^, its disease burden, including a considerable number of non-ICU and ICU patients, was still high in Germany during the second half of 2021, when the delta variant dominated^2^. The transfer of patients was guided by their individual characteristics and the capabilities of the hospitals involved but also undertaken preventively to restore capacities, especially regarding intensive care units (ICU). Several studies analyzed whether the transfer of ICU patients was linked to mortality^5,7,11–13^, with the common result that no adversely effect could be found, with one exception, mentioning even an improved survival for inter-regional transferred ICU patients compared to non-transferred patients during the first COVID-19 wave in France^8^. In addition to mortality, the period of hospitalization is of major interest, as a longer stay may increase shortages of hospital beds, which was also implied by a study without the possibility of verification^8^. A number of studies have addressed the period of hospitalization as secondary outcome^7,8,12^ primarily in ICU patients, and only one of them included non-ICU patients^5^. Recently published data from Germany^6^ demonstrated in great detail the rates and modes of transfer between hospitals of different levels of care in the first wave of COVID-19. However, the study did not address possible links between inter-hospital transfers and outcomes, specifically the period of hospitalization. Due to its implications, the impact of transfers on the period of hospitalization seems worth of investigation and may well supplement the previous findings. Any insight gained in this regard could tell us lessons for improving the management of similar stress situations in the future.

Based on these considerations we analyzed ICU and non-ICU-related transfers in a large dataset from a network of hospitals located in Southern Germany^14,15^. The study period was chosen as that of the 2nd half of 2021, corresponding to the 4th wave of COVID-19 in Germany. This was based on the consideration that COVID-19 was frequent enough at that time to exert considerable stress on the health-care system, but at the same time its therapeutic and resource management had been well established^16,17^, in contrast to previous waves. The specific aims of our study were to compare the clinical characteristics of transferred and non-transferred patients, to determine the associations between transfer and the period of hospitalization or in-hospital mortality, and to identify factors associated with the transfer.

## Methods

### Study population

The data base covered all patients admitted between August 1st 2021 and December 31st 2021 to one of the four acute care hospitals (Rosenheim, Wasserburg, Bad Aibling, Prien am Chiemsee) of the RoMed clinic group in South-East Bavaria, Germany, either as primary admission or after transfer from an external clinic. Patients were required to show a positive PCR test for SARS-CoV-2 upon admission or during their hospital stay. We excluded all patients in whom the positive test was not linked to the admission, especially obstetrical and trauma patients without any COVID-19 symptoms and an incidental positive PCR test. If patients were admitted more than once with a positive PCR test, the first admission was chosen. Moreover, patients of age less than 16 years were excluded, since the clinical characteristics of young patients are likely to differ from those of older patients. The hospitals Rosenheim, Wasserburg, Bad Aibling and Prien am Chiemsee had a capacity of 622, 130, 140 and 140 beds, and a level of care of 2, 1, 1 and 1^18^, respectively. The transfers comprised those in which any of these four hospitals was involved, either as transfer between them, or as transfer to or from a hospital external to the RoMed hospital group. Approval was obtained from the Ethics Committee of the Ludwig-Maximilian-University Munich, Germany (#21–1099; November 22nd, 2021), and all research was conducted in accordance with the Declaration of Helsinki. According to the ethics approval, no written consent from patients was needed from the participants in the retrospective analysis of anonymized data.

### Assessments

The presence of SARS-CoV-2 infection was determined by standardized PCR tests performed upon admission or during hospitalization, using procedures established in the respective clinics^19^. Moreover, requiring a Ct value of at most 30 in order to avoid false-positive tests. Information on anthropometric characteristics, comorbidities (listed in the Supplemental Table S1), treatment limitations as defined previously^15^, medical treatment including low-flow and high-flow oxygen supply, non-invasive ventilation (NIV), ICU admission, intubation, extracorporeal membrane oxygenation (ECMO) and pharmacological treatment (see Supplemental Table S2) was collected by reviewing medical files and records. Low-flow oxygen supply comprised application via Venturi mask/nasal cannula at 2–4 L/min in the great majority of cases, in rare cases up to 15 L/min, high-flow application via nasal cannula (HFNC) or Venturi mask from 30 up to 60 L/min, depending on the patient’s compliance. Complications during the hospital stay comprised the occurrence of bacterial infection, acute kidney injury, bacterial superinfection, pulmonary embolism, and metabolic/electrolyte disorders (see Table 2). Initial vital signs upon admission, specifically heart rate, respiratory rate, blood pressure, oxygen saturation (SpO_2_), were included (see Supplemental Table S2). Moreover, the laboratory parameters determined in the patients’ first examination in the hospital were those listed in the Supplemental Table S2. In addition, blood gas parameters were determined (pH, bicarbonate, arterial pressure of oxygen (PaO_2_)) and used in the computation of the APACHE-II-Score for ICU patients.

Based on the files and medical records it was determined for each patient whether a transfer had occurred due to medical reasons except ECMO, or due to capacity reasons, or due to the need for ECMO. If capacity reasons were explicitly mentioned, these were chosen. The same was true for medical reasons. If no reason was explicitly stated, a medical reason was assumed. There was no instance of both medical and capacity reason for the same patient.

To characterize disease severity and mortality risk in ICU patients, the APACHE-II-Score^20^ was determined, however in simplified form, since information regarding the Glasgow-Coma-Scale (GCS) was not available in most patients. As the GCS has a maximum of 15 points, the maximum value of the simplified score was therefore 56 instead of 71 points. Assuming that upon admission all patients could be categorized as emergency patients, a value of 5 points (maximum value) was chosen in case of a potentially relevant comorbidity (as listed in the Supplemental Table S1). In the absence of these, a value of 0 was chosen.

### Outcomes

#### Period of hospitalization

The period of hospitalization was determined from the patients’ records and files, starting from the date of initial admission to a RoMed hospital and adding up all single length of hospital stays after potential transfer. In case of primary admission to an external clinic, the starting date was that of admission in the external clinic. If this was not known (*n* = 14, mostly due to formal issues), patients were excluded from the analysis of the period of hospitalization. The day of admission was counted as 1 day of period of hospitalization, even if patients had left the hospital at the same day (*n* = 6). The period of hospitalization ended if patients were released from one of the acute care hospitals considered in this study or transferred to a non-acute unit, for example a rehabilitation clinic.

#### Mortality

Mortality was analyzed in terms of in-hospital mortality. This information was available in all patients, but information on patients’ death after final release from the hospitals was not available, primarily due to data-related privacy concerns. In one patient, no follow-up data at all could be collected, therefore this patient (treated with ECMO) was omitted from the analysis.

### Data analysis

Results were described via numbers and percentages, or median values and quartiles, depending on the type of the data. For comparisons of groups, the Mann–Whitney U-test, or the Kruskal–Wallis-test, or the chi-square statistics or Fisher’s exact test were used, depending on the type of data and design. In case of the comparison of more than two groups, post hoc-testing was performed with the Mann–Whitney U-test and appropriate Bonferroni correction. As patients with ICU stay are likely to differ in many clinical respects from patients who did never have an ICU stay, and the need for ICU support could have been an obvious reason for transfer, these two groups were analyzed separately. Linear multiple regression analysis was employed to identify statistically independent correlates of the total length of the period of hospitalization in the two groups. The strategy of the regression analyses and the choice of variables as predictors are described in the Supplement in detail. Statistical significance was assumed for *p* < 0.05. All analyses were performed with the software package SPSS (Version 26, IBM Corp., Armonk, NJ, USA).

### Ethical approval

Approval was obtained from the Ethics Committee of the Ludwig-Maximilian-University Munich, Germany (#21-1099; November 22nd, 2021), and all research was conducted in accordance with the Declaration of Helsinki. According to the ethics approval, no written consent from patients was needed from the participants in the retrospective analysis of anonymized data.

## Results

### Study population

Of 812 patients initially considered, 52 were excluded, since SARS-CoV-2 was judged as not clinically relevant for their hospital stay, consequently 760 patients were included. Their characteristics, treatment and clinical outcomes are shown in Tables 1 and 2, as well as the Supplementary Tables S1 and S2. These tables also contain a comparison of transferred with non-transferred non-ICU and ICU patients. Of the 760 patients, 202 were transferred at least once, and among these 95 to an ICU and 107 to a general ward (see Supplemental Table S3). In addition, 29 patients were transferred twice and 2 patients three times, which was the maximum per patient. All following statistical comparisons of groups refer to the first transfer of a patient.

**Table 1:** Basic characteristics of patients. Numbers and percentages (in parentheses) are given, or median values and quartiles (in parentheses), where appropriate. Comparisons between groups were performed with the Mann–Whitney U-test or the chi-square statistics or Fisher´s exact test, depending on the type of variable and design. *Percentages refer to the respective column depending on available data per category. ^1^ Comparison between non-transferred patients without any ICU stay and transferred patients without any ICU stay. ^2^ Comparison between non-transferred patients with any ICU stay and transferred patients with any ICU stay. If not indicated otherwise, data from all n = 760 patients were available. ICU patients comprise those with ECMO therapy.

	All patients n = 760	Patients without ICU stay, n = 545			Patients with ICU stay, n = 215		
		Non-transferred* n = 458	Transferred* n = 87	*p*-value^1^	Non-transferred n = 100	Transferred n = 115	*p*-value^2^
Sex				0.349			0.085
Female	328 (43.2%)	21 (45.9%)	45 (51.7%)		40 (40.0%)	33 (28.7%)	
Male	432 (56.8%)	248 (54.1%)	42 (48.3%)		60 (60.0%)	82 (71.3%)	
Age (years)	69.0 (53.0–81.0)	69.0 (51.0–82.0)	81.0 (72.0–87.0)	< 0.001	69.0 (52.5–77.0)	62.0 (55.0–73.0)	0.170
Age ≥ 65 years	427 (56.2%)	252 (55.0%)	73 (83.9%)	< 0.001	56 (56.0%)	46 (40.0%)	0.021
Bmi (kg/m^2^) (n = 360)	27.4 (24.2–32.4)	26.4 (23.3–32.0)	26.3 (23.7–31.1)	0.985	28.4 (26.0–34.3)	29.4 (27.5–34.0)	0.537
Living situation (n = 486)				< 0.001			0.653
Alone	79 (16.3%)	41 (13.6%)	22 (38.6%)		8 (13.1%)	8 (12.1%)	
Shared household	361 (74.3%)	228 (75.5%)	29 (50.9%)		50 (82.0%)	54 (81.8%)	
Care-dependent	46 (9.5%)	33 (10.9%)	6 (10.5%)		3 (4.9%)	4 (6.1%)

**Table 2:** In-hospital complications, treatment and clinical outcomes. Numbers and percentages (in parentheses) are given, or median values and quartiles (in parentheses), where appropriate. Comparisons between groups were performed with the Mann–Whitney U-test or the chi-square statistics or Fisher´s exact test, depending on the type of variable and design.

	All patients n = 760	Patients without ICU stay, n = 545			Patients with ICU stay, n = 215		
		Non-transferred* n = 458	Transferred* n = 87	*p*-value^1^	Non-transferred* n = 100	Transferred* n = 115	*p*-value^2^
Complications							
Bacterial infection	169 (22.2%)	63 (13.8%)	22 (25.3%)	0.010	32 (32.0%)	52 (52.0%)	0.051
Acute kidney injury	100 (13.2%)	36 (7.9%)	18 (20.7%)	< 0.001	18 (18.0%)	28 (28.0%)	0.318
Pulmonary superinfection	78 (10.3%)	22 (4.8%)	5 (5.7%)	0.786	17 (17.0%)	34 (34.0%)	0.037
Pulmonary embolism	37 (4.9%)	8 (1.7%)	0 (0.0%)	0.367	14 (14.0%)	15 (15.0%)	0.844
Metabolic/electrolyte disorders	34 (4.5%)	12 (2.6%)	6 (6.9%)	0.052	8 (8.0%)	8 (8.0%)	0.800
Simplified APACHE-II-Score (n = 303)	13.0 (8.0–17.0)	-	-	-	13.0 (8.0–16.0)	13.0 (8.3–18.0)	0.980
≥ 1 Treatment limitation	229 (30.1%)	142 (31.0%)	51 (58.6%)	< 0.001	24 (24.0%)	12 (12.0%)	0.010
Low-flow oxygen	551 (72.5%)	305 (66.6%)	67 (77.0%)	0.060	83 (83.0%)	96 (83.5%)	1.000
High-flow oxygen	195 (25.7%)	36 (7.9%)	7 (8.0%)	1.000	67 (67.0%)	85 (73.9%)	0.295
Non-invasive ventilation	140 (18.4%)	13 (2.8%)	3 (3.4%)	0.729	44 (44.0%)	80 (69.6%)	< 0.001
Invasive mechanical ventilation	79 (10.4%)	-	-	-	16 (16.0%)	63 (63.0%)	< 0.001
ECMO therapy	11 (1.4%)	-	-	-	0 (0.0%)	11 (11.0%)	< 0.001
Hospital stay, total time (days) (n = 744)	9.0 (5.0–17.0)	7.0 (4.0–11.0)	18.0 (11.0–29.0)	< 0.001	12.0 (8.3–18.0)	22.0 (15.0–34.0)	< 0.001
Stay in RoMed hospitals	9.0 (5.0–15.0)	7.0 (4.0–11.0)	18.0 (9.0–30.0)	< 0.001	12.0 (8.3–18.0)	11.0 (8.3–18.0)	0.384
Stay on ICU in a RoMed hospital (n = 208)	7.0 (5.0–14.0)	-	-	-	6.0 (3.0–9.0)	10.0 (6.0–20.0)	< 0.001
Mortality (n = 759)	153 (20.2%)	64 (14.0%)	19 (21.8%)	0.073	31 (31.0%)	39 (34.2%)	0.663

*Percentages refer to the respective column. ^1^ Comparison between non-transferred patients without any ICU stay and transferred patients without any ICU stay. ^2^ Comparison between non-transferred patients with any ICU stay and transferred patients with any ICU stay. ^3^ The simplified APACHE-II-Score was computed for ICU patients only. Information regarding the Glasgow-Coma-Scale (GCS) was not available in many patients, thus the maximum value of the modified score was 56 instead of 71 points, as the GCS has a maximum of 15 points. Moreover, information on the severity of organ dysfunction was not regularly available in the files. Assuming that upon admission all patients could be considered as emergency patients, a value of 5 points (maximum value) was assumed for each patient with a relevant comorbidity. This value was used in the computation of the simplified APACHE-II-Score. In the absence of relevant comorbidities, a value of 0 was used. If not indicated otherwise, data from all n = 760 patients were available. ICU patients comprise those with ECMO therapy.

### Clinical characteristics of transferred versus non-transferred patients

#### Patients without ICU stay

Transferred patients were significantly older than non-transferred patients and more often had the status of living at home alone, while less often living in a shared household (Table 1). The distribution of comorbidities (Supplemental Table 1) also showed differences between groups, with higher prevalence of systemic hypertension, cerebrovascular disease, COPD, kidney disease, malignant disease, hepatic disease, and dementia in transferred compared to non-transferred patients. This was reflected in a significantly higher number of comorbidities in transferred patients. Regarding bacterial infections and acute kidney injury (Table 2), percentages were also higher in the transferred patients. This was also true for the presence of at least one treatment limitation. The percentages of oxygen therapy and non-invasive ventilation did not show significant differences between groups (Table 2), however therapy with corticosteroids or antibiotics was more frequent in transferred compared to non-transferred patients (Supplemental Table S2). Vital signs upon admission did not significantly differ between groups (Supplemental Table S2), and the same applied to most of the laboratory parameters, except for hemoglobin and eGFR, which were lower in the transferred patients, while the levels of troponin, N-terminal pro–B-type natriuretic peptide (NT-proBNP) and sodium were higher (Supplemental Table S2).

#### Patients with ICU stay

The proportion of patients with age ≥ 65 years was higher in non-transferred patients. There were no significant differences regarding the living situation and the frequency of comorbidities, however a tendency towards lower prevalence of COPD and malignant diseases in transferred patients (Supplemental Table S1). Among complications, the proportion of pulmonary superinfection was higher in transferred patients and that of bacterial infections showed a tendency towards higher prevalence (Table 2). Transferred patients showed a lower proportion of treatment limitations, and underwent more often non-invasive and invasive mechanical ventilation and ECMO therapy. There were no significant differences regarding pharmacological therapy (Supplemental Table S2). In transferred patients, the Horovitz-Score was lower, while the levels of glutamate pyruvate transaminase/alanine transaminase (GPT/ALT) and NT-proBNP were higher (Supplemental Table S2).

### Reasons for transfer versus level of care

**Figure 1:**
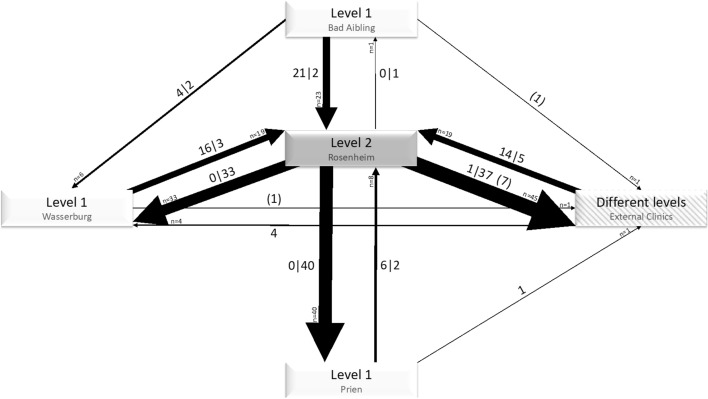
Transfers of patients between hospitals. The diagram also shows the level of care of the hospitals. The thickness of the arrows indicates the number of patients transferred into a particular direction, with the numerical value given at the respective arrowhead. The two numbers at each arrow that are separated by a vertical bar indicate the number of patients transferred due to medical reasons (first number) versus capacity reasons (second number), while the numbers given in parentheses indicate the number of patients transferred due to the need for ECMO therapy. The counts add up to the total number of transferred patients except one patient for whom the reason could not be identified.

The leading reason for the first transfer comprised capacity issues (61.9%), followed by medical reasons (33.2%) and ECMO therapy (4.5%). The flows of transferred patients and the distributions of reasons are delineated in Fig. 1. Transfers due to capacity reasons were mostly directed from the level 2 hospital to the level 1 hospitals, whereas transfers due to medical reasons (except ECMO therapy) primarily involved transfer from level 1 hospitals to the level 2 hospital. Transfers to external clinics were mostly due to the need for ECMO therapy or capacity reasons and mainly involved the level 2 hospital. Accordingly, 91.0% of patients transferred due to medical reasons were upgraded regarding the level of care category, while only 1.5% were downgraded. Conversely, 86.4% of patients transferred due to capacity reasons were downgraded in category, and 8.8% were upgraded. Further details including the clinical units involved can be found in the Supplement and the Supplemental Table S3. This table also provides information on subsequent transfers.

### Period of hospitalization and mortality as outcomes

#### Patients without ICU stay

**Figure 2:**
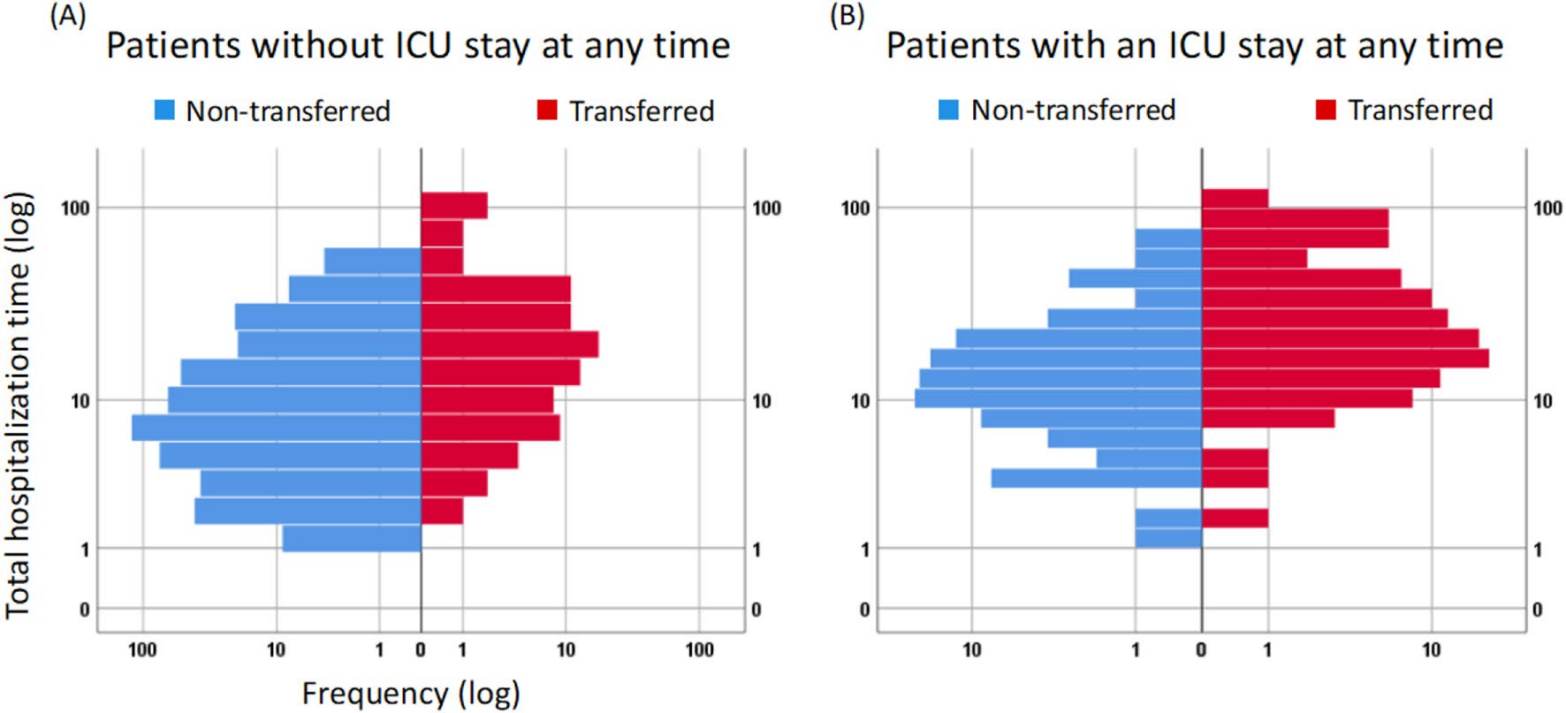
Distribution of total hospitalization time. Results are shown for patients without ICU stay (Panel **A**) and patients with ICU stay (Panel **B**). Absolute numbers are given on the horizontal axis, hospitalization time is shown on the vertical axis. Non-transferred patients are indicated by blue bars, transferred patients by red bars. Please note the logarithmic scale on both axes which was chosen to accommodate for the large range of numbers, hospitalization times and extreme values in their distributions.

Compared to non-transferred patients, both, the period of hospitalization and the time spent in a RoMed hospital, were much longer in transferred patients (Table 2). Figure 2A shows the distribution of the period of hospitalization and illustrates that in non-transferred patients short times were quite frequent, whereas very long times occurred only in transferred patients. Mortality was slightly higher in the transferred patients, but the difference was not statistically significant (Table 2).

#### Patients with ICU stay

The period of hospitalization was nearly twice as long in transferred compared to non-transferred patients (Table 2), and the same was true for the ICU duration in a RoMed hospital. The distribution of the period of hospitalization is illustrated in Fig. 2B. Mortality was very similar in both groups (Table 2).

### Period of hospitalization stratified for influencing factors

**Figure 3:**
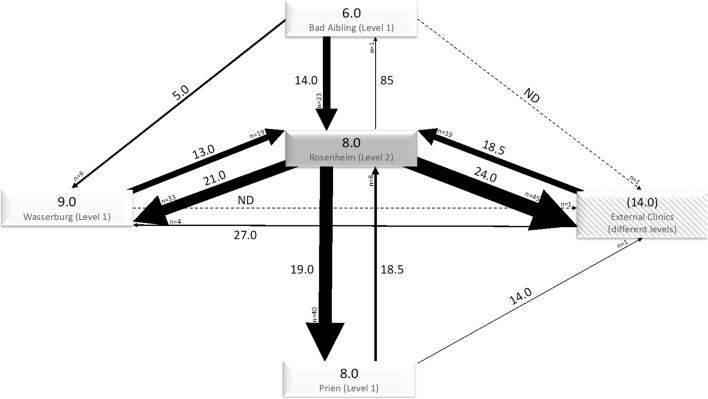
Hospitalization time for transferred and non-transferred patients. The diagram is similar to Fig. 2, again with the thickness of the arrows indicating the number of patients transferred, but with median values of the hospitalization times at each arrow. ND means no data were available; this is also indicated by the dashed lines. The numbers within the rectangles indicate the median value of the hospitalization time for those patients admitted to the respective hospital and never transferred. Regarding the external clinics, the number indicates the median value of hospitalization time after transfer to the external clinics, since we did not have information on patients of these clinics who were never transferred. Patients with ECMO therapy (*n* = 9) and unknown reason for transfer (*n* = 1) are omitted from this diagram.

Figure 3 shows the period of hospitalization in a flow diagram analogous to Fig. 1. Obviously, in most hospitals the period of hospitalization was much shorter in non-transferred patients compared to patients transferred to or from the respective hospital. Moreover, the transfer to a lower level of care was mostly associated with a longer period of hospitalization. The Supplemental Figure S1 shows the data given in Fig. 1 stratified for patients with and without ICU stay, again demonstrating prolonged periods of hospitalization in transferred compared to non-transferred patients.

To identify factors associated with the period of hospitalization, values were stratified for reasons for transfer and level of care of the receiving hospitals (Table 3). Patients transferred due to the need for ECMO therapy are listed separately; their median stay was 40.5 days and thus greater than the median values of all other groups. In the total patient population, the period of hospitalization was significantly (*p* ≤ 0.001 each) associated with the reason for transfer (medical versus capacity) and the level of care of the receiving relative to the discharging hospital.

**Table 3:** Period of hospitalization for subgroups of patients. For comparison, results for the non-transferred patients are repeated from Table 2. Median values and quartiles (in parentheses) are given. n = respective number of patients. In the subgroups of transferred patients shown ECMO patients (n = 8) were excluded from the count, as they represented a special category and their data is given separately. In addition, one patient in whom the reason for transfer could not be identified was excluded from this table. Tests were performed with the Mann–Whitney U-test or the Kruskal–Wallis-test, depending on the number of categories to be compared (2 versus 3). *tested against the complementary group. **Hospitalization times were different (Mann–Whitney U-test with Bonferroni correction, *p* < 0.05) when comparing “upgrade” with “downgrade”. ***Hospitalization times were different (*p* < 0.05) when comparing “same” with “downgrade” and “upgrade” with “downgrade”.

	Period of hospitalization (days)	*p*-value
Transfer for ECMO therapy (n = 8)	40.5 (27.8–77.5)	< 0.001*
Non-transferred patients with available data (n = 558)	8.0 (4.0–13.0)	< 0.001
All transferred patients with available data (n = 176)	19.0 (13.0–30.0)	< 0.001
Reason of transfer		0.001
Medical (n = 53)	14.0 (7.0–25.5)	
Capacity (n = 123)	21.0 (15.0–32.0)	
Level of care of receiving hospital		0.001**
Higher (n = 57)	14.0 (8.0–24.0)	
Same (n = 11)	14.0 (5.0–31.0)	
Lower (n = 108)	21.5 (15.0–32.0)	
Without ICU stay at any time		
Non-transferred (n = 458)	7.0 (4.0–11.0)	< 0.001*
Transferred (n = 81)	18.0 (11.0–29.0)	0.068*
Reason for transfer		< 0.001
Medical (n = 32)	11.0 (6.0–20.0)	
Capacity (n = 49)	21.0 (15.0–32.0)	
Level of care of receiving hospital		< 0.001***
Higher (n = 33)	13.0 (8.0–22.0)	
Same (n = 5)	5.0 (3.0–10.5)	
Lower (n = 43)	22.0 (16.0–32.0)	
With ICU stay at any time (w/o ECMO patients)		
Non-transferred (n = 100)	12.0 (8.3–18.0)	< 0.001*
Transferred (n = 95)	21.0 (14.0–31.0)	0.068*
Reason for transfer		0.396
Medical (n = 21)	17.0 (12.0–33.5)	
Capacity (n = 74)	21.0 (15.0–31.3)	
Level of care of receiving hospital		0.208
Higher (n = 24)	16.0 (11.0–26.3)	
Same (n = 6)	26.0 (14.8–34.0)	
Lower (n = 65)	21.0 (15.0–32.0)	
ICU to ICU transfers (n = 45)	24.0 (15.5–33.5)	0.161*
General ward to ICU transfers (n = 32)	20.0 (13.3–33.5)	0.909*

#### Patients without ICU stay

**Figure 4:**
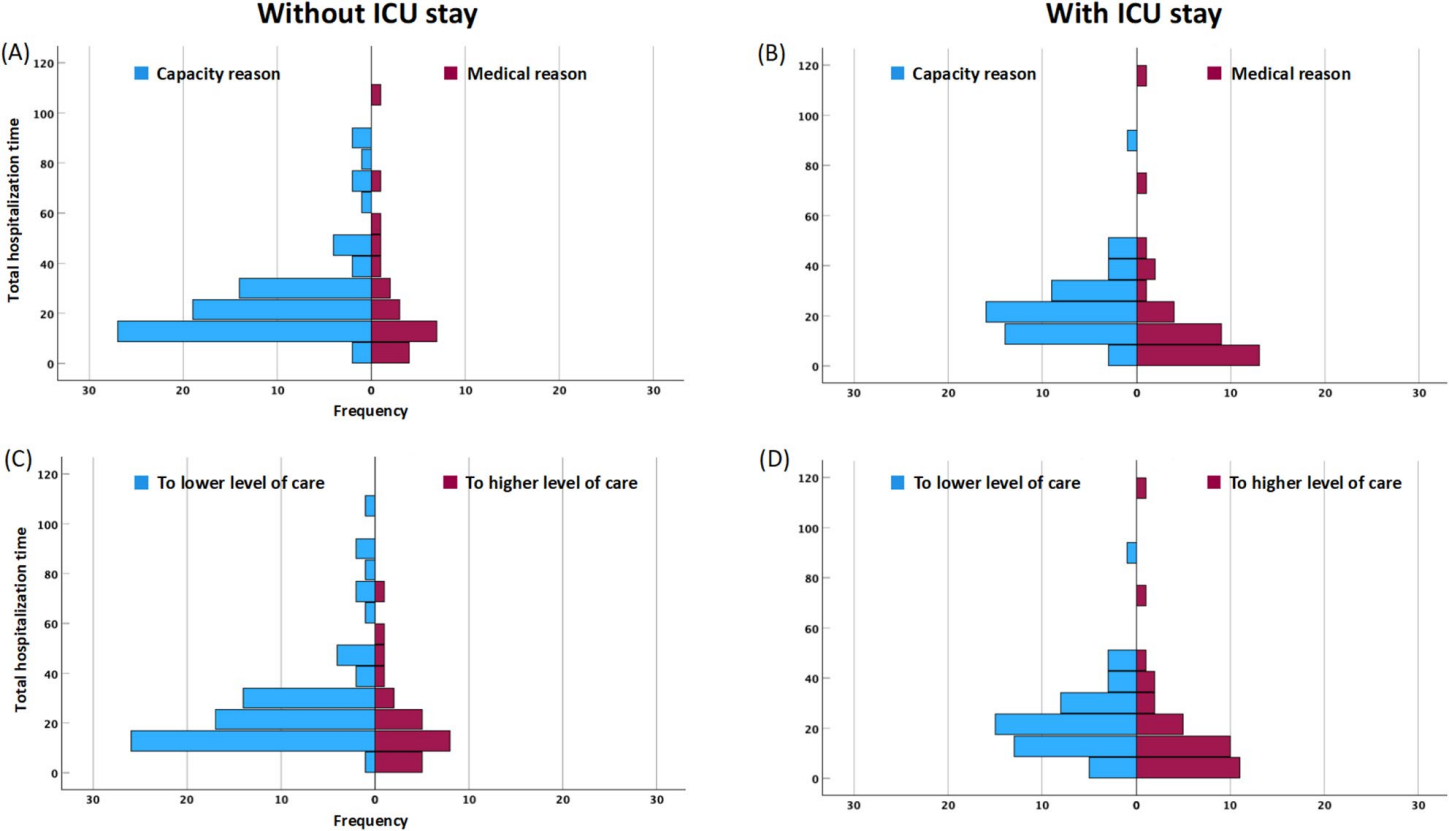
Distribution of the period of hospitalization stratified for the reason for transfer and change of level of care. The upper to panels (**AB**) refer to the reason, the lower two panels (**CD**) to the change in the level of care. The left column (panels **AC**) provides the data for patients without ICU stay, the right column (panels **BD**) for patients with ICU stay. The vertical axes of each panel show the period of hospitalization, the horizontal axes the absolute numbers of patients. Please note that for better comparability the scales are the same. The color code for the respective subgroups is indicated in each panel.

The same pattern as for all patients was found in those without ICU stay, again demonstrating the association with the reason for transfer and the level of care of the receiving hospital (Table 3), with differences between categories larger than in the total group of patients. Patients transferred to a hospital of lower care level or due to capacity reasons showed the by far longest period of hospitalization. The dependence on reasons and change in the level of care was also apparent, when comparing the distributions of the period of hospitalization (Fig. 4A and C, respectively) in the patients without ICU stay.

#### Patients with ICU stay

Differences of the period of hospitalization between categories were much smaller than in patients without ICU and not significantly different. There was also no dependence on the fact whether the transfer occurred from ICU to ICU or from a general ward to an ICU. The lack of dependence on the reason for transfer and the change in the level of care can be clearly seen in Fig. 4B and D, respectively.

### Comprehensive analysis of the relationship between the period of hospitalization and transfer

The results given so far indicate that transfer was associated with a longer period of hospitalization but also with several risk factors and patient characteristics that could influence the stay. To analyze these relationships in detail, we performed multiple linear regression analyses, using clinically plausible predictors of hospital stay. The results are given in the Supplement. Briefly, in the patients without ICU stay, transfer remained significantly (*p* < 0.001) associated with a prolongation of the hospital stay (by 12.7 days) after multiple adjustment for other predictors (see Supplement). In the ICU patients, the prolongation comprised 10.5 days (*p* = 0.048) but was no more significant, if patients who died were excluded (5.8 days, *p* = 0.376).

## Discussion

The present study addressed the association between the inter-hospital transfer of COVID-19 patients and the period of hospitalization or mortality. In accordance with most previous findings , transfer was not significantly linked to mortality. In contrast, the period of hospitalization was robustly associated with transfer, even after considering multiple factors that could have had an influence. These factors comprised clinical characteristics and complications, as well as the fact whether patients living alone at home. Patients without ICU stay showed an average prolongation of the period of hospitalization by more than 12 days, while in-hospital death was associated with a reduction by about 5 days. As reasons for transfer, capacity reasons were slightly more frequent than medical reasons. In patients with ICU stay, the period of hospitalization was again longer in transferred patients by about 12 days, but the result was dependent on the fact whether patients survived, since in-hospital death was associated with an average reduction by about 16 days. In these patients, capacity reasons for transfer were more than three times as frequent as medical reasons, besides ECMO. In all patients, the reasons for transfer were correlated with the change in the hospitals’ level of care after transfer. Capacity reasons were predominantly associated with lowering, and medical reasons with raising of the level. Taken together, these findings indicate that at least in the region and at the time analyzed, COVID-19 patients transferred between hospitals showed a markedly longer period of hospitalization than non-transferred patients did. To which extent the prolongation was due to the transfer per se or due to specific characteristics of the preferentially transferred patients and corresponding physicians’ decisions, cannot be conclusively inferred from our data, despite the consideration of individual risk profiles. Despite this, one should keep in mind the fact that inter-hospital transfers were statistically associated with an increased overall utilization of the health care system.

In-hospital mortality did not significantly depend on the fact whether a transfer had occurred or not, provided that the groups of ICU and non-ICU patients were analyzed separately. The separate analysis was necessary, as ICU patients were relatively more often transferred and at the same time showed a much higher mortality than non-ICU patients. The finding regarding mortality of transferred ICU patients is in line with the great majority of the previous studies on mortality in ICU patients performed in different countries at different periods of time^5,7,8,11–13^, suggesting the validity and comparability of our data.

When focusing on the mortality of non-ICU patients, our results appear novel but compatible with the literature, as in a previous study^5^ no higher mortality of transferred patients was observed, when pooling ICU and non-ICU patients after adjustment for several risk factors. This finding is of interest, as transfer per se can pose a risk especially in ICU patients^21–23^. Similarly, in the non-ICU patients studied by us, we observed only a tendency, which completely disappeared in a multivariable analysis considering risk factors such as age (see Table 1), bacterial infection and acute kidney injury that differed between transferred and non-transferred non-ICU patients (see Table 2). In-hospital death was associated with much greater average reduction of the period of hospitalization in ICU compared to non-ICU patients, which not only underlined the importance of performing separate analyses for these patients but also suggested that ICU patients who did not survive, died relatively early in their hospital stay.

Particularly at times of high burden from COVID-19, inter-hospital transfer of patients was frequent, whereby many of the transfers were due to the need for ICU treatment. However, transfers to general wards also occurred, especially in hotspots as found in many places including the region of the present study in Southern Germany. It may be argued that transfers are trivially associated with prolongation of hospitalization due to the time required for discharge and subsequent readmission; this, however, would account for no more than 2 days. Our study suggests that clinical and social factors could also be involved, besides the additional efforts and time for disinfection, allocation of rooms and similar needs particularly in COVID-19 patients^22^. Indeed, a recently published study^6^ demonstrated a longer time of transportation in COVID-19 compared to other patient, implying a longer total period of hospitalization, if transfer is included, as in our study.

For better understanding of the transfer of COVID-19 patients, we categorized the reasons into medical and capacity reasons, besides ECMO. Our study took advantage from the fact that data could be collected in a network of four acute care hospitals including a dedicated COVID-19 hospital, all of which were experienced in the management of COVID-19 and had detailed and comparable documentation. In studying the impact of transfer, we thus benefited from the availability of a multitude of data describing patients’ characteristics and circumstances of transfer, in addition to the fact that the study comprised a time and location with high numbers of COVID-19 patients and stress on the health-care system.

Regarding the reasons for transfer, a previous study already yielded clues in a comprehensive analysis during the first wave of COVID-19, using the level of care of the hospitals, type of transport vehicle and incidence numbers^6^. Our results demonstrate that the change of level of care associated with transfer was closely linked to its reason suggesting that the change in level is a useful proxy for the reason. The previous study^6^ revealed that in times of highest incidence, transfers towards higher levels dominated, whereas 4 weeks later there was a peak for transfers to lower levels. Using our result, this would imply that initially medical reasons dominated and later capacity reasons played a major role. This interpretation seems plausible from clinical experience, particularly in view of the dynamic situation encountered in the first wave. We did not perform a time series analysis of our data because the case numbers would have been too low to allow for statistically reliable conclusions, but a tentative analysis showed a similar picture as in the previous study^6^. It should also be acknowledged that our study refers to a period when the therapeutic and transfer management of COVID-19 patients was already well established, in contrast to the first wave.

The differences in the periods of hospitalization cannot be understood without consideration of the decision processes of the treating physicians. According to clinical practice, transfer of ICU patients preferentially took place in patients who were hemodynamically stable but expected to need prolonged care, with a likely beneficial outcome. Naturally, ICU patients with either fulminant course or expected rapid recovery were not the primary candidates for transfer. This was reflected in the high proportion of capacity reasons as well as the finding that in the multiple regression analysis death had an extreme shortening effect on the period of hospitalization.

In non-ICU patients, the decision process was more complex and less dictated by medical reasons; instead, capacity reasons dominated. There was an interference with medical reasons in the sense that patients, in whom a prolonged recovery occurred or who were in late stage COVID-19, were probably more often transferred. As suggested by our finding that in non-ICU patients living at home alone (16% of this group) was associated with a period of hospitalization being longer by about 5 days indicated that the management of COVID-19 patients extended beyond acute hospital care. The result was robust in the sense that it was obtained after adjustment for several factors associated with disease severity and the period of hospitalization. Thus, other explanations may be needed. One of them could be that stress situations of the health-care system imply shortage of resources, with the consequence that more time is needed to organize a sufficient degree of care after discharge from the hospital in patients living alone compared to patients living in a shared household or nursing facility. Quarantine requirements may also have interfered with the period of hospitalization, for example if discharge was delayed in order to reduce the risk for transmission of infection in shared households and nursing environments. If this should have been true, the prolongation associated with living alone at home would have been even greater. These considerations suggest that shortages outside the hospital environment may have had repercussions on the time spent in the hospital. The problem may have been further exaggerated by shortages of staff in hospitals and nursing institutions.

When comparing the prevalence of comorbidities between transferred and non-transferred patients, ICU patients did not show significant differences between the two groups, except for tendencies for COPD and malignant diseases being less frequent in transferred patients. There was also no difference in the number of comorbidities. This suggests that in ICU patients these risk factors played a minor role for transfer, in accordance with the observation that most transfers in these patients occurred due to capacity reasons. In contrast, medical reasons appeared to play a relatively greater role in non-ICU patients, corresponding to the fact that the number of comorbidities was greater in transferred compared to non-transferred patients. The number of comorbidities that significantly differed between transferred and non-transferred patients was larger than for ICU patients, but this might be partially due to differences in statistical power. Despite this, it seems plausible that certain comorbidities might have favored a transfer, in particular those that might have required prolonged care. Correspondingly, in non-ICU patients, bacterial infection was associated with a prolongation of the period of hospitalization by about 4 days.

A further factor linked to extended care could be the presence of cerebrovascular diseases and dementia. This assumption is again supported by our data (see Supplemental Table S1), as in line with the increased age, the observations suggested an impaired clinical state in transferred non-ICU patients; this was indicated by the impaired renal function and lower hemoglobin level, as well as the higher need for corticosteroids and antibiotics. As the differences in the prevalence of cardiovascular diseases were minor, the higher levels of troponin and NT-proBNP in transferred non-ICU patients might have been linked to the higher age. In ICU patients, the most obvious finding was the reduction of the Horovitz-Score in the transferred patients, potentially indicating a greater severity of the respiratory impairment.

## Limitations

Our study focused on the period of hospitalization as major outcome and did not analyze the impact of transfer on long-term outcomes. In addition, the determination of the reasons for transfer had to rely on the information given in the files and medical records. If the reason was not explicitly stated, a medical reason was assumed. Thus, we may have underestimated the percentage of transfers due to capacity reasons. Moreover, we had only limited information to compute the APACHE-II-Score, which might have affected the finding that transferred and non-transferred ICU patients did not differ in the simplified score. Although we collected a broad variety of data, we also might have missed clinical factors potentially explaining the differences in the period of hospitalization. The clinical characteristics considered primarily referred to admission but we also included parameters of clinical course, especially complications and treatment modalities. Moreover, the relevant factors might have comprised psychological and social factors such as living alone at home, which we indeed identified as relevant. Due to the complexity of the situation, we primarily aimed at a descriptive analysis and thus supplied explicit p-values not adjusted for the multiplicity of testing, wherever possible. Our result is confined to the second half of 2021 and a limited region of Southern Germany and thus might not be applicable to other regions and times or infection waves, respectively. On the other hand, it was obtained in a standardized and well-experienced hospital environment. Considering, that Germany is known to have a high number of ICU resources compared to many other countries and that despite this fact issues for ICU patients emerged, it could be of interest to compare our findings on the period of hospitalization with those of other countries. The result might depend on the number of available ICU beds (about 25,000 in 2021 in Germany) and total hospital beds (about 484,000 in 2021) relative to a population of about 84 Mio people.

## Conclusion

Based on data from a hospital network located in Southern Germany we studied the impact of inter-hospital transfers of COVID-19 patients in the second half of 2021. While mortality did not differ between transferred and non-transferred patients, this was clearly the case for the period of hospitalization in both ICU and non-ICU patients. The median period of hospitalization was 7 days in non-transferred and 18 days in transferred non-ICU patients. In ICU patients, the respective number were 12 and 21 days. In non-ICU patients, medical versus capacity reasons for transfer were of similar frequency, whereas in ICU patients, capacity reasons were about three times more frequent. The dependence on transfer was maintained after adjustment for multiple confounders. Taken together, the transfer of COVID-19 patients between hospitals was statistically associated with longer periods of hospital stay, corresponding to greater health care utilization, for which however specific patient characteristics and clinical decisions played a role. It remains to be established, to which degree these results depend on specific characteristics of different health care systems.

### Supplementary Information

Supplementary Information.

## Data Availability

The dataset analysed in the current study is not publicly available due to patients’ privacy but available from the corresponding author upon reasonable request.

## Abbreviations

COVID-19: COronaVIrus disease of 2019
SARS-CoV-2: Severe acute respiratory syndrome coronavirus 2
ICU: Intensive care unit
ECMO: Extracorporeal membrane oxygenation
PCR: Polymerase chain reaction
Ct: Cycle threshold
APACHE: Acute physiology and chronic health evaluation
GCS: Glasgow coma scale
ND: No data
BMI: Body mass index
NIV: Non-invasive mechanical ventilation
PAD: Peripheral artery disease
CAD: Coronary artery disease
COPD: Chronic obstructive pulmonary disease
Bpm: Beats per minute
mmHg: Millimeters of mercury
SpO_2_: Peripheral oxygen saturation
CRP: C-reactive protein
eGFR: Estimated glomerular filtration rate
GOT/ALT: Glutamic oxaloacetic transaminase/alanine transaminase
NT-proBNP: N-terminal pro–B-type natriuretic peptide
LDH: Lactate dehydrogenase
IgG: Immunglobulin G
