# Supplementary material for: Period of hospitalization and mortality in transferred versus non-transferred COVID-19 patients: results from Germany
Source: Sci Rep. 2024 Mar 28;14:7338. doi: 10.1038/s41598-024-57272-y (PMC10973428; doi:10.1038/s41598-024-57272-y)
Supplement: Supplementary file 1 — Supplementary Information. [file 41598_2024_57272_MOESM1_ESM.pdf]

Supplement to

Period of hospitalization and mortality in transferred versus non-transferred COVID-19 patients: Results from Germany

Pascal Suski[1], Rudolf A. Jörres[2], Sebastian Engelhardt[3], Kathrin Kahnert[4,5], Katharina Lenherr[1], Andreas Bauer[6], Stephan Budweiser[1]*

*Corresponding author

[1]Department of Internal Medicine III, Division of Pneumology and Respiratory Medicine, RoMed Hospital Rosenheim, Ellmaierstraße 23, 83022 Rosenheim, Germany

[2]Institute and Outpatient Clinic for Occupational, Social and Environmental Medicine, LMU University Hospital, LMU Munich, Comprehensive Pneumology Center Munich (CPC-M), Member of the German Center for Lung Research (DZL), Ziemssenstraße 1, 80336 Munich, Germany

[3]Department of Emergency, RoMed Hospital Rosenheim, Ellmaierstraße 23, 83022 Rosenheim, Germany

[4]Department of Medicine V, LMU University Hospital, LMU Munich, Comprehensive Pneumology Center, Member of the German Center for Lung Research (DZL), Marchioninistraße 15, 80336 Munich, Germany

[5]Pneumological Center, MediCenter Germering, Hartstraße 52, 82110 Munich, Germany

[6]Institute for Anesthesiology and Surgical Intensive Care Medicine, RoMed Hospital Rosenheim, Ellmaierstraße 23, 83022 Rosenheim, Germany

SUPPLEMENTAL RESULTS

Categorization of transfers

The Supplemental Table S3 demonstrates that the leading reason for the first transfer comprised capacity issues (61.9%), followed by medical indications (33.2%), whereas ECMO therapy (4.5%) played a minor role. For comparison, the table also provides data on the 31 cases of subsequent transfers, again showing the high number of capacity reasons. Among the transfers to an ICU, the majority occurred from ICU to ICU compared to general ward to ICU transfers. If there was a transfer to a general ward, this occurred in only 3 of 107 patients as transfer from an ICU, and in the remaining patients as transfer from a general ward. This pattern was maintained in subsequent transfers.

Regarding the level of care of the hospitals, 55.0% of transfers involved transfer to a lower level, 38.6% to a higher level, and 6.4% to the same level. Again, the pattern was preserved in subsequent transfers.

We additionally aimed to describe the transfers between hospitals in more detail, as four RoMed hospitals and several other hospitals were involved in the treatment of COVID-19 patients. The Supplemental Table S2 demonstrates that most transfers occurred within the RoMed hospital group, followed by transfers to external clinics. Only 11.4% of transfers occurred as transfers from external clinics into the RoMed group.

Comprehensive analysis of the relationship between period of hospitalization and transfer

In order to disentangle the relationship between patients characteristics, risk factors, transfer and hospital stay and reveal whether the difference associated with transfer would remain robust when including these additional factors, we employed multiple linear regression analysis, using clinically plausible predictors that could have an influence on hospital stay.

Patients without ICU stay

Using transfer as single predictor, this corresponded to a mean prolongation by 12.5 days (p<0.001). The whole set of predictors that were additionally tested comprised variables from Tables 1, 2 and 3, as well as the Supplemental Table S1, as follows: age, living situation (alone, shared household, care-dependent), complications (bacterial infection, acute kidney injury, pulmonary superinfection, pulmonary embolism, metabolic/electrolyte disorders), the reason for transfer (medical vs capacity), the number of comorbidities, and death. When introducing the additional predictors age, complications (Table 2), living situation (Table 1), the number of comorbidities (Supplemental Table S1) and death, transfer remained highly significant (p<0.001), with a mean (95%CI) effect of 12.7 (9.6; 15.9) days on the period of hospitalization. This was also associated with bacterial infection (3.9 (0.8; 7.1) days; p=0.014), living alone (5.1 (2.1; 8.0) days; p=0.001) and death (-4.8 (-8.1; -1.4) days; p=0.007). A similar result for the effect of transfer was obtained when patients who died (n=83) were excluded from the analysis.

If the reason of transfer, either medical or capacity-related, was added as predictor, transfer was still significantly linked to the period of hospitalization, with a similar estimate and confidence interval, while the reason was not significant. As the distribution of time was skewed (see Figure 1), the analysis was repeated with a hospital stay winsored at 60 days, as well as with its logarithm. This confirmed transfer as being significantly linked to the period of hospitalization (p<0.001 each).

Patients with ICU stay

ECMO patients were excluded from this analysis due to their very specific conditions compared to patients without ECMO. Transfer as single predictor corresponded to a mean prolongation by 11.8 days (p<0.001). The set of additional predictors and the strategy of analysis followed the same approach as in the patients without ICU stay. When using the same additional predictors as in the non-ICU group, transfer remained significant (p=0.048), with a mean (95%CI) effect of 10.5 (0.1; 21.0) days on hospital stay. The period of hospitalization was not associated with any of the other predictors except death (-15.8 (-29.7; -1.8) days; p=0.027). If patients who died (n=63) were excluded from the analysis, transfer was no more significant (p=0.376), with a mean effect of 5.8 (-7.4; 18.9) days. If the reason of transfer was added as predictor, transfer was again significantly linked to hospital stay, with a similar estimate and confidence interval as above, while the reason was not significant. When the analysis was repeated with a hospital stay winsored at 60 days, or with its logarithm, transfer was confirmed as a significant prolongator of stay (p<0.05 each). These findings indicated the major role of death regarding the relationship between transfer and hospital stay in patients with ICU stay.

	All patients (%) n=760	Non-transferred without ICU stay (%)* n=458	Transferred without ICU stay (%)* n=87	p-value[1]	Non-transferred with any ICU stay (%)* n=100	Transferred with any ICU stay (%)* n=115	p-value[2]
**Systemic hypertension**	343 (45.1%)	200 (43.7%)	51 (58.6%)	0.013	43 (43.0%)	49 (42.6%)	1.000
**Peripheral arterial disease (PAD)**	26 (3.4%)	13 (2.8%)	6 (6.9%)	0.100	4 (4.0%)	3 (2.6%)	0.707
**Heart failure**	88 (11.6%)	57 (12.4%)	15 (17.2%)	0.229	10 (10.0%)	6 (5.2%)	0.203
**Coronary artery disease (CAD)**	88 (11.6%)	57 (12.4%)	6 (6.9%)	0.198	12 (12.0%)	13 (11.3%)	1.000
**Atrial fibrillation**	149 (19.6%)	91 (19.9%)	21 (24.1%)	0.386	21 (21.0%)	16 (13.9%)	0.206
**Cerebrovascular disease**	91 (12.0%)	58 (12.7%)	20 (23.0%)	0.018	9 (9.0%)	4 (3.5%)	0.149
**Thrombosis/Embolism**	40 (5.3%)	25 (5.5%)	8 (9.2%)	0.216	4 (4.0%)	3 (2.6%)	0.707
**Obesity**	194 (25.5%)	112 (24.5%)	15 (17.2%)	0.167	34 (34.0%)	33 (28.7%)	0.461
**Diabetes mellitus type 2**	143 (18.8%)	70 (15.3%)	19 (21.8%)	0.153	28 (28.0%)	26 (22.6%)	0.431
**COPD**	53 (7.0%)	29 (6.3%)	12 (13.8%)	0.024	9 (9.0%)	3 (2.6%)	0.070
**Asthma**	48 (6.3%)	25 (5.5%)	10 (11.5%)	0.052	6 (6.0%)	7 (6.1%)	1.000
**Other lung disease***	11 (1.4%)	10 (2.2%)	1 (1.1%)	1.000	0 (0.0%)	0 (0.0%)	-
**Kidney disease**	272 (35.8%)	155 (33.8%)	42 (48.2%)	0.015	38 (38.0%)	37 (32.2%)	0.392
**Malignant disease**	85 (11.2%)	40 (8.7%)	19 (21.8%)	0.001	17 (17.0%)	9 (7.8%)	0.058
**Autoimmune disease**	37 (4.9%)	22 (4.8%)	8 (9.2%)	0.120	3 (3.0%)	4 (3.5%)	1.000
**Hepatic disease**	29 (3.8%)	13 (2.8%)	7 (8.0%)	0.027	3 (3.0%)	6 (5.2%)	0.508
**Depression/psychiatric disorders**	74 (9.7%)	45 (9.8%)	13 (14.9%)	0.182	9 (9.0%)	7 (6.1%)	0.446
**Dementia**	87 (11.4%)	51 (11.1%)	25 (28.7%)	<0.001	7 (7.0%)	4 (3.5%)	0.354
**State of immunosuppression**	65 (8.6%)	34 (7.4%)	10 (11.5%)	0.201	11 (11.0%)	10 (8.7%)	0.648
**Number of comorbidities**	2 (1-4)	2 (1-4)	3 (2-5)	<0.001	2 (1-4)	2 (1-3)	0.130
**0**	149 (19.6%)	99 (21.6%)	7 (8.0%)	0.003	19 (19.0%)	24 (20.9%)	0.864
**≥1**	611 (80.4%)	359 (78.4%)	80 (92.0%)	0.003	81 (81.0%)	91 (79.1%)	0.864
**≥3**	334 (43.9%)	192 (41.9%)	54 (62.1%)	<0.001	44 (44.0%)	44 (38.3%)	0.407
**≥7**	33 (4.3%)	18 (3.9%)	9 (10.3%)	0.026	5 (5.0%)	1 (0.9%)	0.099

Supplemental Table S1: Comorbidities. *Percentages refer to column. Comparisons between groups were performed with Fisher´s exact test, or the Mann-Whitney U-test, depending on the type of variable. *Percentages refer to the respective column. [1] Comparison between non-transferred patients without any ICU stay and transferred patients without any ICU stay. [2] Comparison between non-transferred patients with any ICU stay and transferred patients with any ICU stay. Data were available in all 760 patients and percentages refer to this number. ICU patients comprise those with ECMO therapy

	All patients	Patients without ICU stay			Patients with ICU stay		
	n=760	Non-transferred* n=458	Transferred* n=87	p-value[1]	Non-transferred n=100	Transferred n=115	p-value[2]
**Vital signs**							
Heart rate (bpm), (n=722)	85 (75-98)	83 (73-95)	80 (71-92)	0.112	90 (80-107)	92 (79-110)	0.672
Mean arterial pressure (mmHg), (n=742)	95.3 (85.7-103.7)	95.0 (85.8-103)	93.5 (83.6-103.0)	0.351	95.7 (84.6-102.9)	96.7 (87.8-104.8)	0.345
Respiratory rate (per minute), (n=589)	20 (18-25)	20 (17-23)	20 (18-22)	0.783	24 (20-30)	24 (20-30)	0.958
SpO_2 (in %), (n=705)	94 (91-96)	95 (93-97)	95 (93-96)	0.389	91 (83-95)	92 (85-94)	0.639
Horovitz-Score, (n=549)	277 (194-330)	303 (241-358)	309 (255-338)	0.935	216 (137-294)	170 (97-252)	0.006
**Laboratory parameters**							
Lactate (mmol/L), (n=546)	1.1 (0.8-1.5)	1.0 (0.8-1.5)	1.0 (0.8-1.3)	0.362	1.2 (0.9-1.6)	1.3 (1.0-1.8)	0.104
CRP (mg/L), (n=728)	5.5 (1.8-11.4)	3.8 (1.3-9.5)	4.4 (1.4-9.6)	0.486	10.6 (4.4-16.8)	9.7 (5.8-17.7)	0.863
Procalcitonin (mg/L), (n=550)	0.17 (0.09-0.5)	0.15 (0.08-0.48)	0.18 (0.09-0.5)	0.168	0.25 (0.12-0.50)	0.18 (0.11-0.40)	0.170
Hemoglobin (g/L), (n=744)	13.8 (12.3-14.9)	13.8 (12.3-14.9)	12.9 (10.9-14.0)	<0.001	14.1 (12.8-15.2)	14.4 (12.8-15.3)	0.256
Platelets (/nl), (n=737)	202 (153-263)	202 (155-258)	184 (141-241)	0.096	206 (151-260)	207 (156-281)	0.736
Leukocytes (/µL), (n=743)	6.7 (5.0-9.3)	6.5 (4.7-8.9)	6.9 (5.1-9.1)	0.626	7.2 (5.6-10.7)	7.4 (5.2-11.1)	0.668
Sodium (mmol/L), (n=729)	136 (133-139)	136 (133-139)	138 (134-140)	0.004	135 (132-138)	135 (132-138)	0.822
Potassium (mmol/L), (n=701)	4.0 (3.7-4.4)	4.0 (3.7-4.4)	4.1 (3.8-4.4)	0.121	4.0 (3.7-4.5)	4.0 (3.6-4.4)	0.807
eGFR (ml/min), (n=723)	72 (45-90)	73 (46-90)	57 (37-80)	0.004	69 (40-95)	76 (51-95)	0.256
GOT/ALT (u/l), (n=716)	28 (18-45)	26 (17-42)	24 (16-34)	0.081	32 (19-48)	39 (27-56)	0.007
NT-proBNP (pg/ml), (n=444)	401 (113-1812)	328 (85-1777)	732 (288-1986)	0.024	675 (155-3089)	282 (90-1267)	0.030
LDH (u/L), (n=608)	315 (229-449)	285 (215-380)	298 (213-387)	0.622	412 (296-544)	479 (335-645)	0.054
Troponin (ng/l), (n=602)	16.7 (8.5-38.1)	13.9 (7.9-36.1)	22.3 (12.0-49.2)	<0.001	19.5 (9.9-49.7)	19.7 (10.3-36.2)	0.766
D-Dimers (ng/ml), (n=598)	460 (247-1076)	418 (230-980	540 (302-1074)	0.219	514 (283-1143)	574 (246-2096)	0.694
**Pharmacological therapy**							
Remdesivir (n=760)	215 (28.3%)	0 (0.0%)	0 (0.0%)	-	0 (0.0%)	0 (0.0%)	-
IgG (n=760)	47 (6.2%)	28 (6.1%)	9 (10.3%)	0.163	5 (5.0%)	5 (4.3%)	1.000
Corticosteroids (n=647)	551 (85.2%)	282 (78.6%)	73 (90.1%)	0.019	85 (91.4%)	111 (97.4%)	0.068
Antibiotics (n=752)	384 (51.1%)	175 (38.8%)	47 (54.0%)	0.009	73 (73.7%)	89 (77.4%)	0.632
Anticoagulation therapy (n=740)	690 (93.2%)	398 (90.7%)	84 (96.6%)	0.088	96 (97.0%)	112 (97.4%)	1.000
Platelet aggregation inhibitor (n=754)	144 (19.1%)	85 (18.8%)	24 (27.6%)	0.079	17 (17.0%)	18 (15.7%)	0.854

Supplemental Table S2: Vital signs and laboratory values as well as pharmacological therapy measures in the different subgroups of patients. sPO_2=peripheral oxygen saturation, CRP=C-reactive protein, LDH=lactate dehydrogenase, GPT/ALT=glutamate pyruvate transaminase/alanine transaminase, eGFR=estimated glomerular filtration rate computed from creatinine levels in blood, NT-proBNP= N-terminal pro–B-type natriuretic peptide, IgG=immunoglobulin G. Numbers and percentages (in parentheses) are given, or median values and quartiles (in parentheses), where appropriate. Comparisons between groups were performed with the Mann-Whitney U-test or Fisher´s exact test, depending on the type of variable. *Percentages refer to the respective column. [1] Comparison between non-transferred patients without any ICU stay and transferred patients without any ICU stay. [2] Comparison between non-transferred patients with any ICU stay and transferred patients with any ICU stay. ICU patients comprise those with ECMO therapy

	First transfer n=202	Subsequent transfers* n=31
**Reasons of transfer**		
Medical indication (except ECMO) (%)	67 (33.2%)	3 (9.7%)
Capacity issue** (%)	125 (61.9%)	26 (83.9%)
Unknown (%)	1 (0.5%)	0 (0.0%)
ECMO therapy (%)	9 (4.5%)	2 (6.5%)
**Clinical units involved**		
General ward to General ward (%)	104 (51.5%)	13 (41.9%)
General ward to ICU (%)	39 (19.3%)	3 (9.7%)
ICU to ICU (%)	56 (27.7%)	10 (32.3%)
ICU to General ward (%)	3 (1.5%)	5 (16.2%)
**Level of care of the receiving hospital**		
Higher (%)	78 (38.6%)	8 (25.8%)
Same (%)	13 (6.4%)	1 (3.2%)
Lower (%)	111 (55.0%)	22 (71.0%)
**Transfer in relation to RoMed hospital group**		
Within RoMed (%)	130 (64.4%)	20 (64.5%)
From RoMed to external (%)	49 (24.3%)	10 (32.3%)
From external to RoMed (%)	23 (11.4%)	0 (0.0%)
From external to external (%)	0 (0.0%)	1 (3.2%)

Supplemental Table S3: Causes and characteristics of transfers. Please note that in the present study only the first transfer was analyzed. Subsequent transfers are shown in this table to demonstrate that their percentages were small compared to those of initial transfers. *Two patients were transferred 3 times, which was the maximum number of transfers for a patient. **Includes back transfers to initial hospital (n=7)

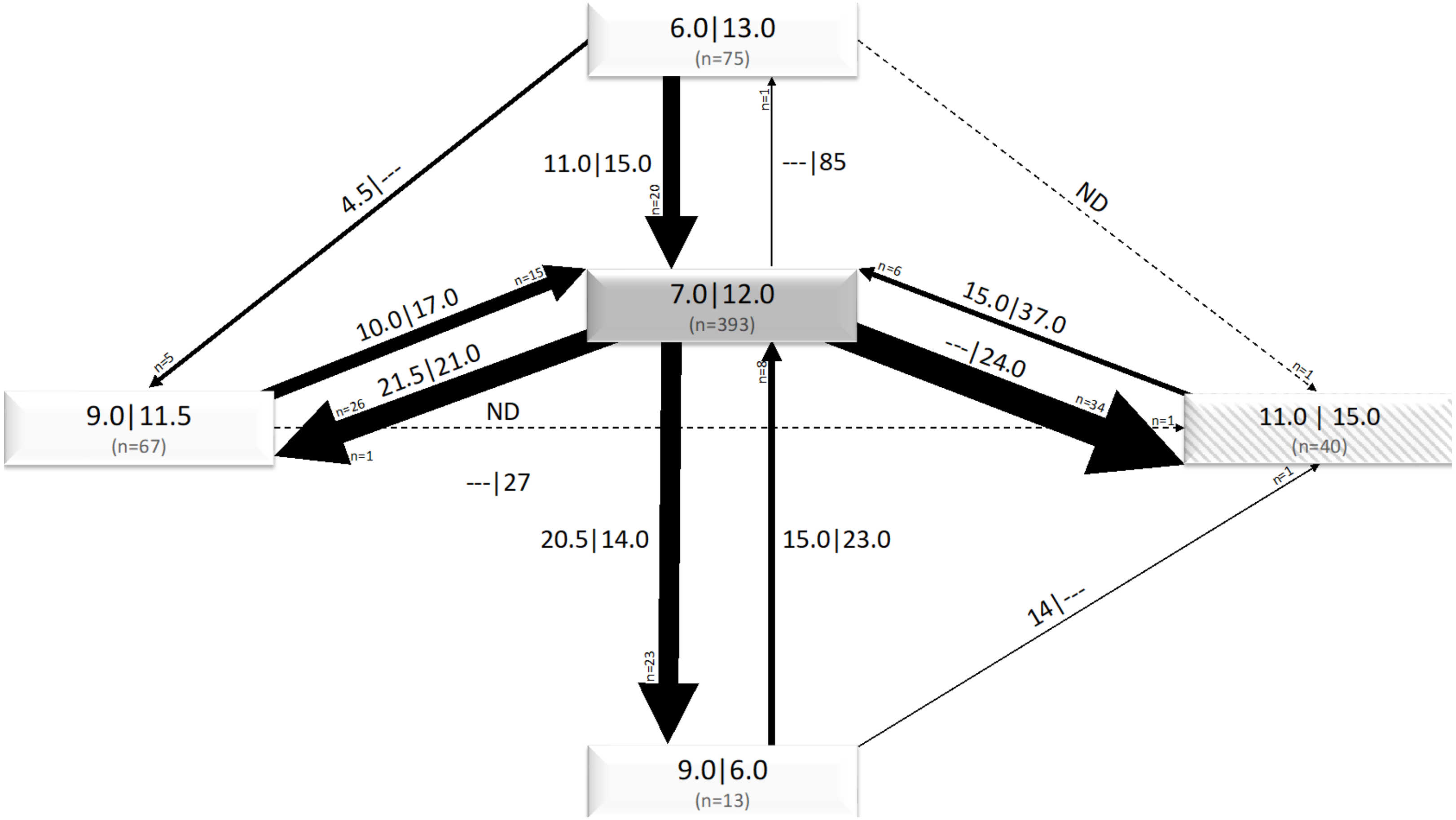

Supplemental Figure S1: Hospitalization times stratified for non-ICU versus ICU patients. The diagram is similar to Figure 3, again with the thickness of the arrows indicating the number of patients transferred between two hospitals and with median values of the hospitalization times at each arrow. Extending Figure 3, hospitalization times are given separately for patients without ICU stay at any time (number before the vertical bar) and patients with any ICU stay (number after the vertical bar). Regarding external clinics, the median value of the hospitalization time after transfer to the external clinics is given. Patients with ECMO therapy (n=9) and unknown reason for transfer (n=1) are omitted from this diagram
